# Apolipoprotein L1 is a tumor suppressor in clear cell renal cell carcinoma metastasis

**DOI:** 10.3389/fonc.2024.1371934

**Published:** 2024-04-12

**Authors:** Linh Nguy-Hoang Le, Cheolwon Choi, Jae-A. Han, Eun-Bit Kim, Van Ngu Trinh, Yong-June Kim, Seongho Ryu

**Affiliations:** ^1^ Soonchunhyang Institute of Medi-bio Science (SIMS), Soonchunhyang University, Cheonan, Republic of Korea; ^2^ Department of Integrated Biomedical Science, Soonchunhyang University, Cheonan, Republic of Korea; ^3^ Department of Urology, Chungbuk National University Hospital, Cheongju, Republic of Korea; ^4^ Department of Urology, Chungbuk National University College of Medicine, Cheongju, Republic of Korea

**Keywords:** APOL1, renal cell carcinoma, metastasis, tumor suppressor, Akt, EMT, focal adhesion, miRNA

## Abstract

The 5-year survival rate of kidney cancer drops dramatically from 93% to 15% when it is metastatic. Metastasis constitutes for 30% of kidney cancer cases, in which clear cell renal cell carcinoma (ccRCC) is the most prominent subtype. By sequencing mRNA of ccRCC patient samples, we found that apolipoprotein L1 (APOL1) was highly expressed in tumors compared to their adjacent normal tissues. This gene has been previously identified in a large body of kidney disease research and was reported as a potential prognosis marker in many types of cancers. However, the molecular function of APOL1 in ccRCC, especially in metastasis, remained unknown. In this study, we modulated the expression of APOL1 in various renal cancer cell lines and analyzed their proliferative, migratory, and invasive properties. Strikingly, APOL1 overexpression suppressed ccRCC metastasis both *in vitro* and *in vivo*. We then explored the mechanism by which APOL1 alleviated ccRCC malignant progression by investigating its downstream pathways. APOL1 overexpression diminished the activity of focal adhesive molecules, Akt signaling pathways, and EMT processes. Furthermore, in the upstream, we discovered that miR-30a-3p could inhibit APOL1 expression. In conclusion, our study revealed that APOL1 play a role as a tumor suppressor in ccRCC and inhibit metastasis, which may provide novel potential therapeutic approaches for ccRCC patients.

## 1 Introduction

According to the SEER database, the 5-year survival rate of kidney cancer is 93% when it is confined to primary site, but dramatically drops down to 15% if it metastasizes to distant parts of the body (1). Notably, kidney cancer is the top ten most common cancers found in males, incidence and mortality rates are 7.8 and 3.0 for every 100,000 males, respectively (2). Clear cell renal cell carcinoma (ccRCC), characterized by loss of chromosome 3p and VHL mutations (3), accounts for 70% of all renal malignancies and is associated with poor survival rates (4). It is important to note that VHL loss by itself does not lead to ccRCC nor triggers distant metastasis (5, 6). Discovering the mechanism behind further cellular dysregulation is critical to understand ccRCC tumorigenesis.

In ccRCC, apolipoprotein L1 (APOL1) is transcriptionally upregulated in a distinct metastatic tumor cell cluster and is associated with poor survival (7). This gene has been shown to be highly expressed in tumor tissues (8–13) and is found to be a potential prognosis marker for many types of cancers via bioinformatic analyses (14–23). Apolipoprotein L gene family is only found in a few higher primates and human beings, and APOL1 is the only secreted member due to its N-terminal signal peptide (24). APOL1 is secreted into the serum by the liver, bound to high-density lipoproteins, complexed to IgM, and is expressed for intracellular transport in several organs (25). Their structures and functions are similar to the Bcl‐2 gene family (B3-only proteins) (26). The molecular functions of APOL1 include chloride channel activity, lipid binding, and other biological processes (27–29). APOL1 is also known to play a role as an oncogene in pancreatic cancer (8). In the Human Protein Atlas database, APOL1 notably has the highest RNA expression level in many kidney cancer cell lines compared to other cancer types (30). The role of APOL1 in ccRCC metastasis remains unknown.

The critical process to metastasis may include mechanisms such as epithelial to mesenchymal transition (EMT) and the expression status of focal adhesion molecules. To invade, resist apoptosis, and to disseminate, carcinoma cells must lose their epithelial phenotypes, detach from epithelial sheets, while gaining the mesenchymal characteristics, which is known as the EMT. In EMT, the decreased expression of E-cadherin, a marker of epithelial cells, and upregulation of N-cadherin, a marker of mesenchymal cells, are known to regulate invasion and metastasis. Moreover, some pleiotropically acting transcriptional factors orchestrate the EMT, such as Snail and Slug (31). Focal adhesions are the contact sites of cell cytoskeleton to extracellular matrix (ECM) through molecules such as integrins. One hallmark of focal adhesions is the clustering of integrins, which affects the organization of the cytoskeleton underlying the plasma membrane. Focal adhesion kinase (FAK) is a key cytoplasmic non-receptor tyrosine kinase that play a key role in cell adhesion and migration process for cancer metastasis (32).

By sequencing mRNA of ccRCC patient samples, we found that APOL1 was highly expressed in renal tumors compared to adjacent normal tissues. The suppression of APOL1 induced the activation of focal adhesion proteins, Akt, and its downstream targets, and initiated the EMT process. These results proposed that the role of APOL1 is as a tumor suppressor to inhibit tumor dissemination, which may provide novel potential therapeutic approaches against renal cancer metastasis.

## 2 Materials and methods

### 2.1 Human patient samples and RNA and microRNA sequencing

Human specimens were collected after obtaining informed consent in accordance with the Declaration of Helsinki and was approved by Institutional Review Board of Chungbuk National University Hospital.

Total RNA and microRNA (miRNA) from patient samples were extracted by miRNeasy Mini Kit (Qiagen) according to the manufacturer’s instructions and was sent to Macrogen (South Korea) for sequencing and analysis.

### 2.2 *In silico* analysis

The expression of genes in renal cancer stages and survival analyses of 20 candidate genes in The Cancer Genome Atlas Program - Kidney renal clear cell carcinoma dataset (TCGA-KIRC) were screened by UALCAN (https://ualcan.path.uab.edu) (33). The signaling pathways were examined by Geneset Enrichment Analysis (GSEA) version 4.3.2 (34), and gene enrichment was analyzed by ConsensusPathDB (http://cpdb.molgen.mpg.de) (35). The Pearson correlation between APOL1 and microRNA candidate in our sequencing data and TCGA-KIRC dataset were performed using GraphPad Prism 9 version 9.3.0 and ENCORI platform (https://rnasysu.com/encori/panMirCoExp.php), respectively (36). The survival curve of APOL1 and miR-30a were generated by using the Kaplan-Meier plot (https://kmplot.com/analysis/index.php?p=service&cancer=pancancer_rnaseq) (37).

### 2.3 Cell culture

HK-2, A-498 and Caki-1 cell lines were purchased from the Korea Cell Bank (South Korea). 786-O cell line was purchased from American Type Culture Collection (USA). All cell lines were cultured at 37°C and 5% CO_2_ in RPMI 1640 medium (10-040-CV, Corning), except for A-498 cells which were cultured in DMEM (10-013-CV, Corning). All media were supplemented with 10% fetal bovine serum (35-015-CV, Corning) and Penicillin Streptomycin (15140-122, Gibco).

### 2.4 Lentiviral transfection

APOL1 shRNA plasmids (V3SH11240-230184401, V3SH11240-229063063, Dharmacon), APOL1 overexpression plasmid (RC217000L3, Origene) were co-transfected with lentiviral packaging vectors (pMD2G and pSPAX2) to HEK293T cells using Lipofector-pMAX (Aptabio, South Korea). The target cells were transduced by the harvested supernatants. The stable cell lines were generated by selection with puromycin.

### 2.5 microRNA transfection

Negative control miRNA and miR-30a (Bioneer, South Korea) were transfected to primary renal cancer cells using Lipofectamine RNAiMAX (Invitrogen). The cells were incubated for 3 days.

### 2.6 RNA extraction and quantitative PCR

Total RNA from cell lysate were extracted by Ribospin II (314-150, Gene All) according to the manufacturer’s instructions. The total amount of RNA was measured by NanoDrop 2000 Spectrophotometer (Thermo Scientific). cDNAs were synthesized by ReverTra Ace qPCR RT Kit (FSQ-101, Toyobo) using the T100 Thermal Cycler (Bio-Rad). Real-time qPCR was performed using SYBR Green Realtime PCR Master Mix (QPK-201, Toyobo) with the CFX96 Real-Time System (Bio-Rad). The primers for APOL1 and β-actin used were APOL1-Forward: 5’-AAGTAAGCCCCTCGGTGACT-3’, APOL1-Reverse: 5’-GAGCTCATCTGCCTCATTCC-3’, β-actin-Forward: 5’-CTCCTTAATGTCACGCACGAT-3’, and β-actin-Reverse: 5’-CATGTACGTTGCTATCCAGGC-3’.

Total RNA and miRNA from tumors were extracted by miRNeasy Mini Kit (Qiagen) according to the manufacturer’s instructions. The total amount of RNA was measured by NanoDrop 2000 Spectrophotometer (Thermo Scientific). miRNAs were reverse transcripted by TaqMan MicroRNA Reverse Transcription Kit (4366597, Applied Biosystems) with miR-30a-3p-RT-primer: 5’-GTCGTATCCAGTCCAGGGACCGAGGACTGGATACGACGCTGC-3’ using the T100 Thermal Cycler (Bio-Rad). Real-time qPCR was performed using TaqMan Universal Master Mix II, no UNG (4440040, Applied Biosystems) with the CFX96 Real-Time System (Bio-Rad). The primers and probe for miR-30a-3p were miR-30a-3p-Forward: 5’-AGCCGCTTTCAGTCGGATGTTT-3’, miR-30a-3p-Reverse: 5’-TCCAGGGACCGAGGA-3’, miR-30a-3p-Probe: 5’-(FAM)-CTGGATACG ACGCTGC-(BHQ1)-3’ (38). The relative level of miR-30a-3p was normalized to RNU48 (assay ID: 001006, Applied Biosystems).

### 2.7 Protein extraction and western blotting

Total protein from cell lysates were extracted by RIPA Lysis and Extraction Buffer (89900, Thermo Scientific) supplemented with Halt Protease Inhibitor Cocktail (78410, Thermo Scientific) and Halt Phosphatase Inhibitor Cocktail (78420, Thermo Scientific). Protein concentrations were measured by Pierce BCA Protein Assay Kit (23225, Thermo Scientific).

For western blotting, the protein samples were mixed with 5X Laemmli loading buffer, and were heated at 95°C for 10 minutes. The ladder and the samples were loaded and resolved on polyacrylamide gel, and were transferred to a PVDF membrane (IPVH00010, Merck). Then the protein membranes were blocked in 5% skim milk for 1 hour at room temperature, and incubated with primary antibody at 4°C overnight. The following antibodies were used: Actin (A2103) from Sigma-Aldrich (USA); APOL1 (ab169952) and Kindlin 1 (ab68041) from abcam (USA); p-Akt (4060), Akt (3063), p-NFκB (3033), NFκB (8242), p-GSK3β (5558), GSK3β (4818), E-cadherin (3195), N-cadherin (4061), Snail (3879), Slug (9585), Integrin αV (4711), Integrin β3 (4702), p-FAK (8556), α-actinin (6487), p-Talin (5426), GAPDH (2118) from Cell Signaling (USA); Vinculin (sc-25336) from Santacruz (USA). Blots were washed with TBS/T three times, followed by an incubation with the secondary Ab (anti Rabbit or anti Mouse) (1:5000) for 1 hour at room temperature (RT). Blots were again washed with TBS/T and developed with the Clarity Max Western ECL Substrate (Bio-Rad). The protein bands were visualized by Chemidoc system (Bio-Rad).

### 2.8 Proliferation assay

Cells were stained with CellTrace CFSE reagent (C34554, Invitrogen) and were incubated for 0, 1, 3, 5 days. Each day, cells were harvested and fixed with 4% paraformaldehyde. Analysis was completed using an FACS Canto II Flow Cytometer (BD Life Sciences, USA) with 488−nm excitation and a 530/30-nm bandpass emission filter. The results were analyzed by FlowJo software version 10 (BD Life Sciences).

### 2.9 Wound healing assay

The cells were seeded on a 6-well plate at 80% confluency. After attaching overnight, a scratch was made by using the 200 µL tip. Images of four random microscopic fields under a light microscope (bright field) were taken at 0 hour and indicated hours with a 10X objective lens. The percentage of wound healing was calculated by TScratch program version 1.0 (ETH Zürich, Switzerland).

### 2.10 Migration and invasion assay

For transwell experiments, 100 µL of 0.4 mg/mL Matrigel (356230, Corning) and 750 µL of complete media (10% FBS) were respectively loaded into the upper chamber and lower chamber of Transwell with 8.0 µm Pore Polycarbonate Membrane Insert (3422, Corning), and incubated for 2 hours in the CO_2_ incubator. After 2 hours, 1 x 10^5^ cells suspension in serum free media were added onto Matrigel-coated cell culture insert and incubate for indicated hours (16h, 18h or 24h). Then, the medium in upper chamber was removed and the insert was washed twice with PBS. Cells were fixed with 4% paraformaldehyde for 2 min at RT, and were washed twice with PBS. Cells were then permeablized with 100% methanol for 20 min at RT, and washed twice with PBS. After that, cells were stained with 0.4% crystal violet and incubated at room temperature for 15 min, and washed twice with PBS. Non-invaded cells were scrapped off with cotton swabs. Invasive cells were counted in four random microscopic fields under the Leica DMi8 microscope (bright field) with a 20X objective lens. For migration assay, the same protocol was performed without Matrigel-coated on the insert.

### 2.11 *In vivo* metastasis assays

The mice experiment protocol was approved by the Institutional Animal Care and Use Committee of Soonchunhyang University (SCH20-0049) and performed in accordance with the Animal Facility of Soonchunhyang Institute of Medi-Bio Science guidelines. 12-week-old male BALC/c nude mice (Nara Biotech, South Korea) were housed in specific pathogen free conditions. The mice were intravenously injected via the tail vein with 2 x 10^6^ cells in 100 μL of 1X PBS of control or APOL1 overexpressing Caki-1 cells. After 8 weeks, these mice were euthanized by carbon dioxide inhalation, and their lungs were perfused and dissected for visual inspection of the metastatic nodules. The lungs were fixed in 4% paraformaldehyde for 24 hours and then were processed and embedded into a paraffin block. Paraffin sections (5 μM) were mounted on microscope slides and stained with Harris hematoxylin (YD Diagostics, South Korea) and eosin (BBC Bichemical, USA), and were imaged with Leica DMi8 microscope (bright field) with a 20X and 40X objective lens. 7-week-old male BALC/c nude mice (Nara Biotech, South Korea) were subcutaneously injected on the right flank with 10^7^ cells in 100 μL of 1X PBS of A-498, 786-O and Caki-1 cells. After 8 weeks, these mice were euthanized, and their tumors were collected for subsequent analysis. For immunohistochemistry, the tumors were fixed in 4% paraformaldehyde for 24 hours and then were processed and embedded into a paraffin block. Paraffin sections (5 μM) were mounted on microscope slides and proceeded with primary antibody APOL1 (sc-390440, Santa Cruz) and counterstained with Harris hematoxylin (YD Diagostics, South Korea). Images were taken by Leica DMi8 microscope (bright field) with a 20X objective lens.

### 2.12 Statistical analysis

Each experiment was performed in triplicates. Statistical analyses were performed using GraphPad Prism 9 version 9.3.0. All tests were two-tailed unpaired t tests. The p-value <0.05 was considered to be statistically significant.

## 3 Results

### 3.1 APOL1 is highly expressed in clear cell renal cell carcinoma

**Figure 1:**
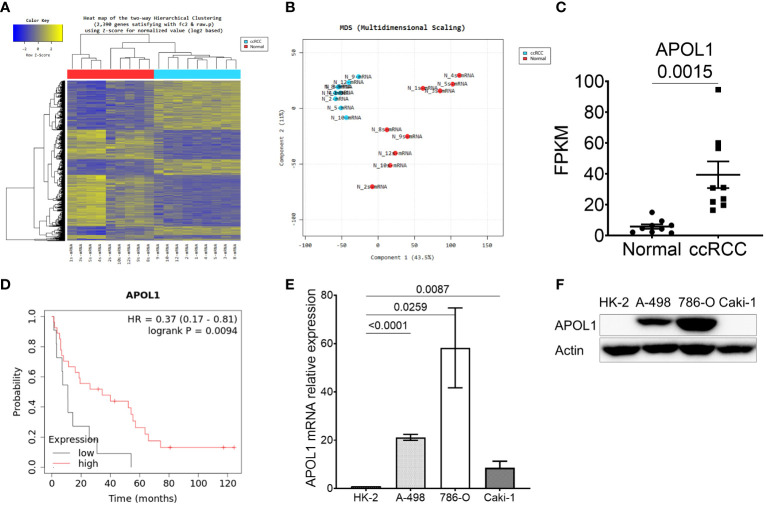
APOL1 is highly expressed in clear cell renal cell carcinoma. **(A–C)** Heat map of hierarchical clustering **(A)**, multidimensional scaling **(B)** and APOL1 mRNA expression **(C)** in RNA-seq of 9 patient ccRCC tumors compared to their adjacent normal tissues. **(D)** Kaplan-Meier survival analysis based on the APOL1 expression at stage 4 and grade 4 in the TCGA-KIRC dataset. **(E, F)** qRT-PCR analysis of APOL1 mRNA **(E)** and western blot analysis of APOL1 protein **(F)** in A-498, 786-O (primary cancer) and Caki-1 (metastatic) cells compared to HK-2 (normal) cells. Actin served as an internal control.

To characterize the molecular mechanisms related to malignant properties of ccRCC, we first performed RNA sequencing (RNA-seq, GSE252600) on 9 pairs of tumors and adjacent normal tissues, with 3 of them having metastatic sites (**Table 1**). The hierarchical clustering heatmap and the multidimensional analysis of the RNA-seq revealed the distinguished expression profiles of tumor from the normal tissues. The tumors were grouped together rather than with their normal adjacent tissues (**Figures 1A, B**). There were 2390 differentially expressed genes (DEG) obtained from the RNA-seq analysis, and among them 459 candidate genes overlapped with the TCGA-KIRC dataset (**Supplementary Figure S1A**). Among the top 20 upregulated genes that were highly expressed in ccRCC compared to normal tissues (**Supplementary Figure S1B**), *TGFBI*, *PLOD2*, *TMEM45A*, *FCGR1C*, *HIST1H2BH*, *CCL5* and *APOL1* were closely correlated to poor survival rate in ccRCC patients. Furthermore, *TGFBI*, *PLOD2* and *APOL1* had clear tumor progression patterns in the TCGA-KIRC dataset (**Figure 1C**, **Supplementary Figures S1C, D**). In an effort to identify which of the three genes we should investigate in, we also sequenced for differential expressions in microRNA expression (GSE252629), to see which gene was the most regulated. We overlapped differentially expressed miRNAs of tumors and adjacent normal tissues with miRNAs targeting these 3 genes. *APOL1* and *TGFBI* were targeted by 28 and 24 miRNAs, respectively, whereas *PLOD2* were targeted by 13 miRNAs (data not shown). We focused on the novelty of APOL1 as a potential cancer marker whose low expression strongly associated to poor survival rate at metastatic stage of ccRCC (**Figure 1D**), considering that TGFBI had already been identified as oncogene in RCC (39).

**Table 1:** The stage and metastatic status of 9 ccRCC human samples.

Sample	pT	pN	pM	Stage	Furhman’s grade	Metastatic site
1	2a	0	0	2	1	
2	1a	0	0	1	1	
3	3a	0	0	3	2	
4	1a	0	0	1	2	
5	1a	0	0	1	2	
8	3a	1	0	3	3	
9	2a	0	1	4	3	neck
10	2a	0	0	2	3	
12	3a	1	1	4	3	lung

After realizing the potential of APOL1 as a candidate marker, we then examined its expression in various renal cell lines. We utilized immortalized proximal tubule epithelial cell line from normal adult human kidney HK-2, clear cell renal cell carcinoma cell lines A-498 and 786-O, and the metastatic cell line Caki-1. Both APOL1 transcript and protein levels were not detected in HK-2 but were robustly expressed in primary cancer cells and retained less expression in the metastatic cells (**Figures 1E, F**). To mimic the condition of primary and metastatic stages of ccRCC, we subcutaneously injected the primary cancer A-498 and 786-O cells and metastatic Caki-1 cells to nude mice, respectively. As a result, APOL1 was highly expressed in tumors of primary cancer cells and lowly expressed in tumor of metastatic cells (**Supplementary Figures S1E, F**). Furthermore, moderate level of *APOL1* were observed in ccRCC patients at late stages (**Supplementary Figure S1G**). These observations were consistent with the fluctuating expression of APOL1 protein in ccRCC from Clinical Proteomic Tumor Analysis Consortium (CPTAC) (**Supplementary Figure S1H**).

### 3.2 miR-30a-3p is downregulated in ccRCC and suppresses APOL1 expression

**Figure 2:**
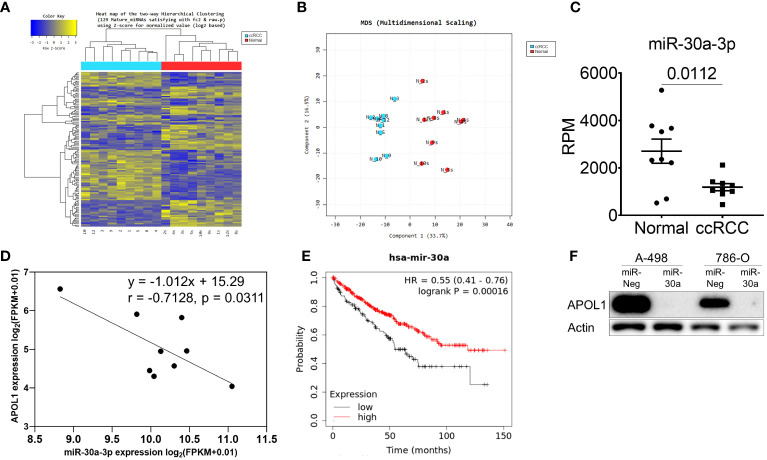
miR-30a-3p suppresses APOL1 expression. **(A–C)** Heat map of hierarchical clustering **(A)**, multidimensional scaling **(B)** and miR-30a-3p expression **(C)** in miRNA-seq of 9 patient ccRCC tumors compared to their adjacent normal tissues. **(D)** Pearson correlation analysis between miR-30a-3p and *APOL1* in patient ccRCC tumors. **(E)** Kaplan-Meier survival analysis based on the miR-30a expression in the TCGA-KIRC dataset. **(F)** Western blot analysis of APOL1 protein in control and miR-30a transfected A-498 and 786-O cells. Actin served as an internal control.

To further investigate the miRNA that could regulate APOL1 expression and ccRCC metastasis, we analyzed small RNA sequencing data (miRNA-seq) of 9 patient samples. Hierarchical clustering heatmap and the multidimensional scaling of miRNA-seq revealed the distinguished expression profiles of the tumor and normal tissues (**Figures 2A, B**). There were 103 differently expressed miRNAs, and among them, 28 miRNAs were predicted to target the 3’UTR region of *APOL1* by TargetScan, where 16 were upregulated and 12 were downregulated miRNAs (**Supplementary Figure S2A**). Due to the high expression of APOL1 in ccRCC, we focused on the miRNAs that had attenuated expression in ccRCC (**Supplementary Figure S2B**), and therefore examined the correlation between *APOL1* and these 12 downregulated miRNAs. We found that miR-30a-3p expression was negatively correlated with *APOL1* expression, and this was statistically significant in both miRNA-seq and the TCGA-KIRC dataset (**Figures 2C, D**, **Supplementary Figures S2C, D, E, F**). In *in vivo* model, the expression of miR-30a-3p was low in tumors of primary cancer cells and high in tumor of metastatic cells, which negatively correlated with APOL1 expression (**Supplementary Figure S2G**). Moreover, the low level of miR-30a-3p in ccRCC (**Figure 2C**, **Supplementary Figures S2H, I**) was associated with poor survival rates (**Figure 2E**). To validate whether the miR-30a-3p targeting 3’UTR of *APOL1* regulated its expression *in vitro*, we transfected this miRNA to the high APOL1 expression renal cells, A-498 and 786-O (**Supplementary Figure S2J**). As expected, APOL1 protein expression was reduced (**Figure 2F**). Furthermore, we treated A-498 cells with the top 3 significantly downregulated miRNAs. As a result, these miRNAs did not specifically suppress APOL1 expression in renal cancer cells as the effect of miR-30a-3p (**Supplementary Figure S2K**). In conclusion, we found that the miRNAs and RNA worked in cohort to actively regulate ccRCC, and miR-30a-3p regulated *APOL1* expression, which is in turn a potential hallmark of ccRCC.

### 3.3 APOL1 suppresses ccRCC metastasis *in vitro* and *in vivo*

**Figure 3:**
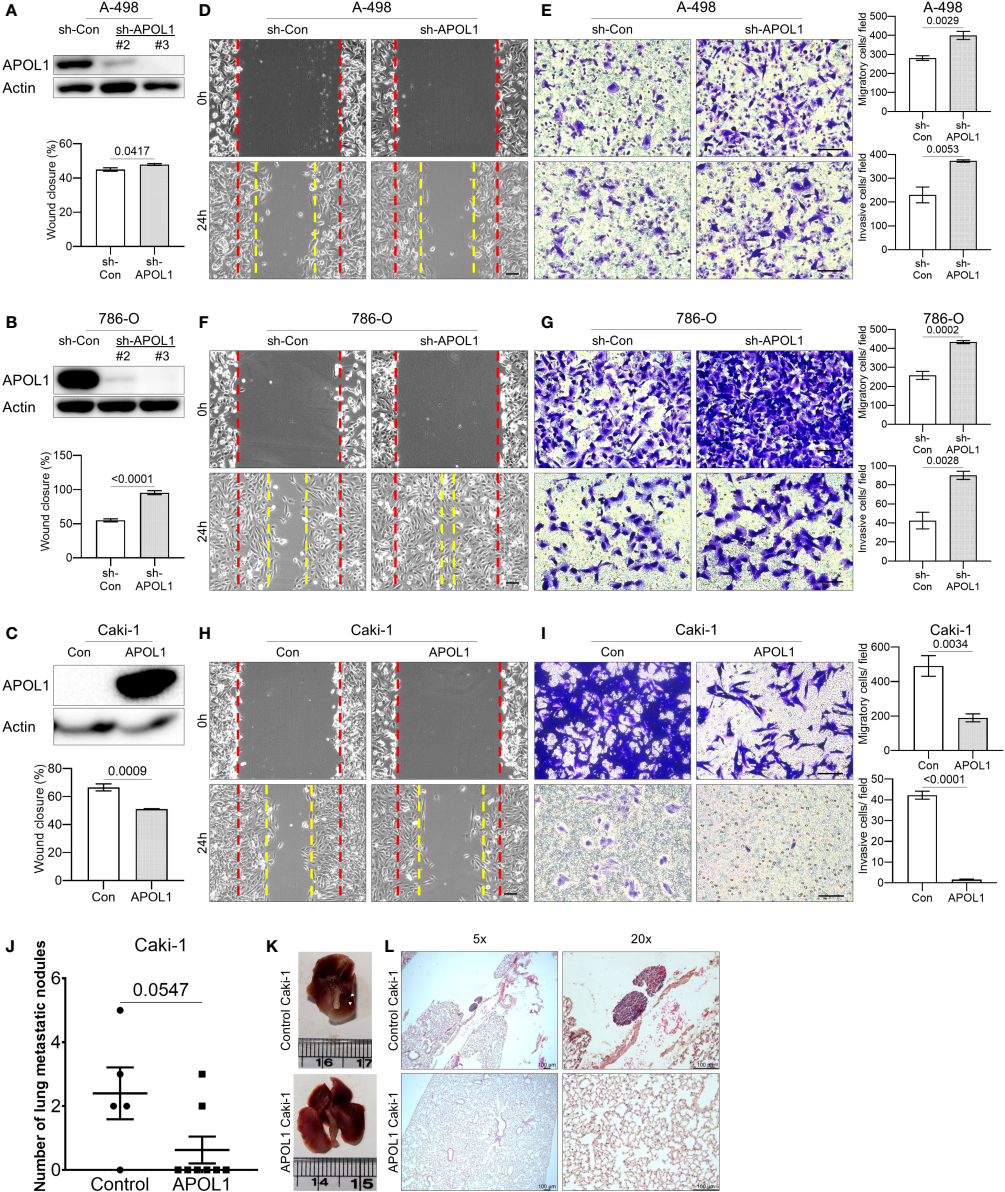
APOL1 overexpression in renal cell carcinoma reduces the metastasis *in vitro* and *in vivo*. **(A–C)** Western blot analysis of APOL1 protein in control and APOL1 knockdown A-498 cells **(A)**, APOL1 knockdown 786-O cells **(B)** and APOL1 overexpressing Caki-1 cells **(C)**. Actin served as an internal control. **(D, F, H)** Wound healing analysis of control and APOL1 knockdown A-498 cells **(D)**, APOL1 knockdown 786-O cells **(F)** and APOL1 overexpressing Caki-1 cells **(H)**. Scale bar, 100 µM. **(E, G, I)** Transwell migration and invasion analysis of control and APOL1 knockdown A-498 cells **(E)**, APOL1 knockdown 786-O cells **(G)** and APOL1 overexpressing Caki-1 cells **(I)** cells. Scale bar, 100 µM. **(J, K, L)** Number of lung metastatic nodules **(J)**, representative image of the dissected lung **(K)** and H&E staining of lung **(L)** in mice intravenously injected with control and APOL1 overexpressing Caki-1 cells. Scale bar, 100 µM.

To assess the role of APOL1 in tumor progression, we generated a stable knockdown of APOL1 in the primary cancer A-498 and 786-O cells and overexpressed APOL1 in the metastatic Caki-1 cells by lentiviral transfection. The renal cancer cells were efficiently knocked down and overexpressed at both transcript and protein levels (**Figures 3A–C**, **Supplementary Figure S3A**). The knockdown and overexpression of APOL1 did not affect the cell proliferation rate (**Supplementary Figure S3B**). Interestingly, the results from the wound healing and transwell assays showed that APOL1 knockdown promoted cell migration and invasion (**Figures 3D–G**). In coherence with these findings, APOL1 overexpression obstructed cell motility (**Figures 3H, I**).

To verify the function of APOL1 expression in metastatic progression *in vivo*, we intravenously injected the APOL1 overexpressing Caki-1 cells into the tail vein of nude mice. In line with the *in vitro* results, the number of lung metastatic nodules in the mice injected with APOL1 overexpressing Caki-1 cells were reduced compared to its control plasmids (**Figures 3J–L**). There was no difference in the weight between control and experimental mouse groups (**Supplementary Figure S3C**). Taken together, we found that APOL1 was critical in the progression of ccRCC, but was shown to block metastasis at later stages of cancer.

### 3.4 Suppression of APOL1 facilitates FAK activation and EMT via the Akt signaling pathway

**Figure 4:**
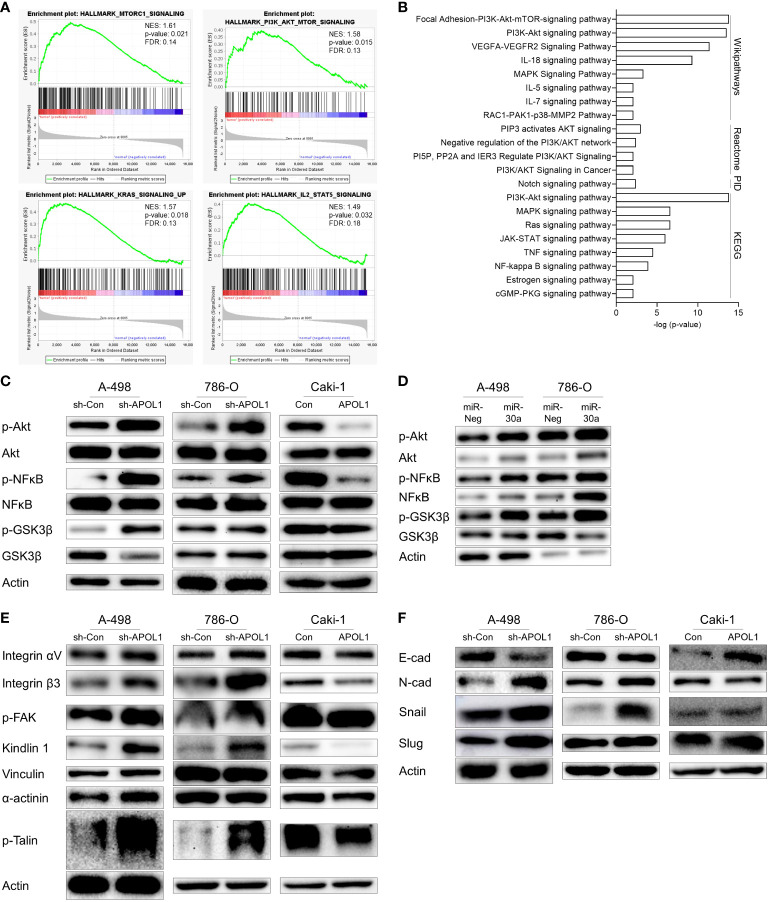
APOL1 suppression activates Akt signaling pathway in clear cell renal cell carcinoma. **(A)** GSEA of RNA-seq data from patient ccRCC tumors compared to their adjacent normal tissues. **(B)** Gene enrichment analysis of 212 upregulated genes in patient ccRCC tumors. **(C, D)** Western blot analysis of Akt, NFĸB, GSK3β protein in control and APOL1 knockdown/overexpressing renal cells **(C)**, and miR-30a transfected A-498 and 786-O cells **(D)**. **(E)** Western blot analysis of focal adhesion proteins in control and APOL1 knockdown A-498 cells, APOL1 knockdown 786-O cells and APOL1 overexpressing Caki-1 cells. **(F)** Western blot analysis of EMT markers in control and APOL1 knockdown A-498 cells, APOL1 knockdown 786-O cells and APOL1 overexpressing Caki-1 cells. Actin served as an internal control.

To gain insights into the mechanism by which APOL1 ameliorated renal malignant progression, we investigated the tumorigenic pathways by GSEA and gene enrichment analyses of 212 upregulated genes in ccRCC. As a result, Akt signaling pathway was shown to be the most significantly activated pathway by analysis with Wikipathways, Reactome, PID, KEGG database, among several other pathways in ccRCC. Namely, focal adhesion-Akt signaling pathway, negative regulation of the PI3K/Akt network, NF-kappa B signaling pathway were shown to be regulated (**Figures 4A, B**). The top terms for GO analysis were the intracellular functions of APOL1 rather than its secreted function, and was shown to be involved in regulation of cellular process and binding functions (**Supplementary Figure S4**).

To determine whether APOL1 dependent Akt pathway could be involved in cell migration of tumor cells, we checked the Akt pathway in the APOL1 knockdown or overexpressing cells. Akt phosphorylation and the downstream targets including nuclear factor kappa light chain enhancer of activated B cells (NFκB) and glycogen synthase kinase 3 (GSK3β) were enhanced in the APOL1 knockdown A-498 and 786-O cells and downregulated in the APOL1 overexpressing Caki-1 cells (**Figure 4C**). Consistent with our findings, APOL1 suppression by miR-30a activated the Akt signaling pathway (**Figure 4D**).

Elevation of many integrin molecules may trigger metastasis in renal cell carcinoma (40, 41), moreover, integrins also activate Akt via FAK, paxillin, and integrin-linked kinase (42). Because APOL1 is known to interact with integrin αVβ3 to activate integrins in podocytes to dysregulate of actin cytoskeleton (43), we checked the expression of several focal adhesion proteins in APOL1 knocked down and overexpressing cells. In agreement with the increased cell migration in APOL1 knockdown cells, focal adhesion proteins integrin αVβ3, p-FAK, kindlin 1, vinculin, α-actinin, and p-Talin was upregulated. Consistent with these findings, APOL1 overexpression downregulated these focal adhesion proteins (**Figure 4E**).

We hypothesized that the EMT could also influence the migration and invasion of tumor cells. Thus, we examined the EMT related protein expression in APOL1 knockdown or overexpressing cells. Mesenchymal marker N-cadherin and transcriptional repressors of E-cadherin (Snail and Slug) were upregulated while epithelial marker E-cadherin was downregulated in APOL1 knockdown cells. On the other hand, APOL1 overexpression inhibited EMT and showed increased E-cadherin (**Figure 4F**).

In summary, suppression of APOL1 activated tumorigenic pathways, especially that of Akt, in which FAK contributed to increase tumor aggressiveness through the expression of adhesion molecules and EMT processes.

## 4 Discussion

ccRCC is the most prominent subtype of kidney cancer, where inactivation of VHL tumor suppressor gene is regarded as the governing event for progressing through HIF transcription factor activation in conventional ccRCC. APOL1, found as a tumor suppressor in our study, is one of the upregulated downstream genes of HIF (44). Similarly, transcriptome sequencing analysis of ccRCC against adjacent normal tissues in our research demonstrated that *APOL1* was highly expressed in the cancer samples with high mortality rates. Furthermore, adenosine to inosine RNA editing events is notably found in the 3’UTR region of *APOL1* across 33 cancer types from TCGA dataset (45). This event leads to the upregulation of APOL1, and is associated with poor overall survival of lung adenocarcinoma (46). This is how APOL1 expression is increased to promote renal cancer progression through cell metabolic alterations (47).

In our study, the mRNA expression of APOL1 is increased according to the stages of cancer, however, the protein level is dropped at the later stages (**Supplementary Figures S1C, G, H**). This phenomenon infers that APOL1 may be modulated during its transcriptional or translational processes to be reduced in its expression in later cancer stages. We found that miR-30a-3p targets APOL1 and found evidence that it may affect ccRCC metastasis via the Akt signaling pathway. However, this finding may be contradictory with the previous studies of miR-30a as a tumor suppressor in renal cell carcinoma and other various carcinomas (48–58). Our discovery revealed the connection between miR-30a-3p and APOL1 as the miRNA to be the inhibitor of the tumor suppressor.

One of our important findings was that APOL1 was highly expressed in primary renal cancer cells and retained lower expression patterns in metastatic cells. This was the same as drop APOL1 protein expression in more aggressive tumor grades (**Supplementary Figure S1H**). Consistently, the downregulation of APOL1 promoted EMT and cell motility of non-metastatic cells, whereas the upregulation of APOL1 suppressed cell migration in metastatic cells. These observations correspond to the previous inference that EMT process arise when knocking out APOL1 (47). Altogether, deregulation of APOL1 protein may be a hallmark of advanced renal cancer.

Our functional enrichment analysis showed that the Akt signaling was the major underlying pathway pertaining to ccRCC progression. The PI3K/Akt signaling pathway was reported to be activated in various renal cancer cell lines regardless of the VHL deficiency (59). Hence, the phosphorylation of Akt may inhibit GSK3β in RCC cells by facilitating GSK3β ubiquitination (60). Subsequently, NFκB is constituently activated in the signaling cascades initiated by phosphatidylinositol 3-kinase (PI3K) and Akt (61). Both NFκB activation and GSK3β degradation may lead to Snail or Slug mediated EMT (62–64). Additionally, Akt is the downstream of FAK (65) and integrin αVβ3, and promotes EMT by upregulation of Slug expression in RCC (66). FAK localizes into integrin receptor cell adhesion sites and to the ECM. When activated, it recruits important components of the cytoskeleton, such as kindlin 1, vinculin, α-actinin, and talin to the cell membrane, providing both an anchorage for attachment and transduce intracellular signals (32). Our study revealed that integrin αVβ3 activating FAK plays as an intermediating role between APOL1 and Akt signaling pathway, facilitating EMT. Altogether, APOL1 is an upstream regulator of these signaling pathways which ultimately leads to ccRCC tumor progression.

In pancreatic cancer, APOL1 is known to promote proliferation and inhibit apoptosis (8); however, in renal cell carcinoma, it does not alter proliferation and instead inhibit cell migration and invasion. APOL1 therefore is involved in diverse cellular processes and may have different roles depending on cancer types. Even though APOL1 is thought to be dispensable (67), its risk alleles have been proved to worsen kidney disease via cytotoxic effect on podocytes (68), furthermore, according to our research, APOL1 has a critical role in ccRCC. To further understand the related molecular mechanisms, direct inhibitory interaction between APOL1 and integrin molecules may need to be elucidated.

**Figure 5:**
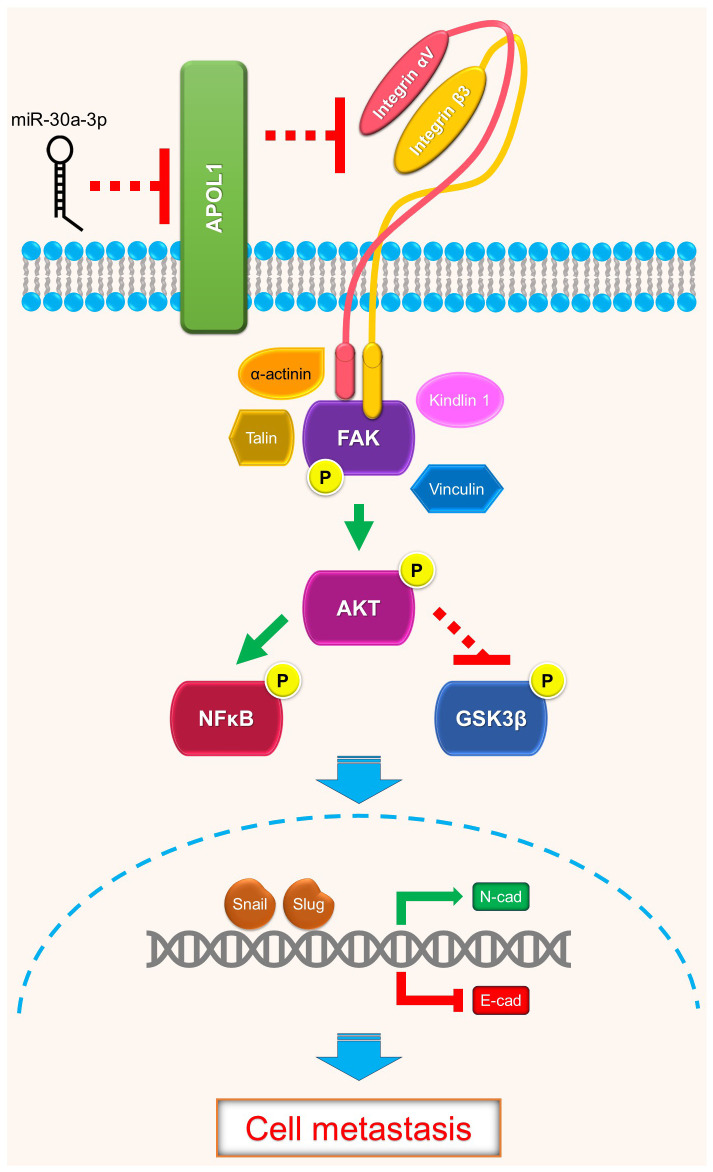
Schematics of APOL1 function in clear cell renal cell carcinoma. APOL1 attenuates renal cancer cell dissemination through FAK/Akt/GSK3β and NFκB-mediated EMT. miR-30a-3p inhibits APOL1 expression.

In summary, renal cancer cells may disseminate to distant organs through activation of focal adhesion proteins and trigger Akt downstream target genes to promote EMT. Our study revealed the intracellular function of APOL1 as a tumor suppressor in renal cancer cell EMT and metastasis through FAK/Akt/GSK3β and NFκB, which may be potentially targeted to control ccRCC metastasis (**Figure 5**).

## Data availability statement

The datasets presented in this study can be found in online repositories. The names of the repository/repositories and accession number(s) can be found below: https://www.ncbi.nlm.nih.gov/geo/, GSE252600, https://www.ncbi.nlm.nih.gov/geo/, GSE252629.

## Ethics statement

The studies involving humans were approved by Institutional Review Board of Chungbuk National University Hospital. The studies were conducted in accordance with the local legislation and institutional requirements. The participants provided their written informed consent to participate in this study. The animal study was approved by Institutional Animal Care and Use Committee of Soonchunhyang University (SCH20-0049) and performed in accordance with the Animal Facility of Soonchunhyang Institute of Medi-Bio Science guidelines. The study was conducted in accordance with the local legislation and institutional requirements.

## Author contributions

LL: Conceptualization, Data curation, Visualization, Writing – original draft. CC: Conceptualization, Data curation, Investigation, Writing – original draft. J-AH: Funding acquisition, Investigation, Methodology, Validation, Visualization, Writing – review & editing. E-BK: Data curation, Investigation, Writing – review & editing. NT: Conceptualization, Data curation, Investigation, Writing – review & editing. Y-JK: Conceptualization, Funding acquisition, Resources, Writing – review & editing. SR: Conceptualization, Funding acquisition, Project administration, Resources, Supervision, Writing – review & editing.

## Supplementary material

Supplementary Figure S1 APOL1 expression in clear cell renal cell carcinoma. **(A)** Analytical procedure of RNA-seq data from patient ccRCC tumors and their adjacent normal tissues. **(B)** List of top 20 upregulated genes in DEGs between ccRCC tumors and their adjacent normal tissues. **(C)** The survival analysis and expression of 20 upregulated genes by the stages in TCGA-KIRC dataset. **(D)** APOL1 mRNA expression in RNA-seq of 9 patient ccRCC tumors compared to their adjacent normal tissues. **(E)** qRT-PCR analysis of APOL1 mRNA in tumors of A-498, 786-O and Caki-1 cells. Actin served as an internal control. **(F)** Immunohistochemistry of APOL1 expression in tumors of 786-O and Caki-1 cells. **(G)** APOL1 expression by cancer stages in patient ccRCC tumors. **(H)** APOL1 protein expression by cancer grades in Clinical Proteomic Tumor Analysis Consortium (CPTAC) samples of ccRCC.

Supplementary Figure S2 miRNA expression in clear cell renal cell carcinoma. **(A)** Analytical procedure of miRNA-seq data from patient ccRCC tumors and their adjacent normal tissues. **(B)** List of 12 downregulated miRNAs that targeting 3’UTR of *APOL1*. **(C, D)** Pearson correlation analysis between downregulated miRNA candidate and *APOL1* in TCGA-KIRC dataset **(C)** and patient ccRCC tumors **(D)**. **(E)** The inverse correlation between APOL1 and miR-30a-3p in 9 patient ccRCC samples. **(F)** miR-30a-3p expression in RNA-seq of 9 patient ccRCC tumors compared to their adjacent normal tissues. **(G)** qRT-PCR analysis of miR-30a-3p in tumors of A-498, 786-O and Caki-1 cells. RNU48 served as an internal control. **(H, I)** miR-30a-3p expression by cancer stages in TCGA-KIRC dataset **(H)** and patient ccRCC tumors **(I)**. **(J)** Sequences of miR-30a-3p and the potential binding site of miR-30a-3p at the 3’ UTR of *APOL1*. **(K)** Western blot analysis of APOL1 protein in control and miR-138, miR-141, miR-204 transfected A-498 cells. GAPDH served as an internal control.
