# Supplementary material for: Apolipoprotein L1 is a tumor suppressor in clear cell renal cell carcinoma metastasis
Source: Front Oncol. 2024 Apr 12;14:1371934. doi: 10.3389/fonc.2024.1371934 (PMC11045967; doi:10.3389/fonc.2024.1371934)
Supplement: Supplementary Figure S1 — APOL1 expression in clear cell renal cell carcinoma. (A) Analytical procedure of RNA-seq data from patient ccRCC tumors and their adjacent normal tissues. (B) List of top 20 upregulated genes in DEGs between ccRCC tumors and their adjacent normal tissues. (C) The survival analysis and expression of 20 upregulated genes by the stages in TCGA-KIRC dataset. (D) APOL1 mRNA expression in RNA-seq of 9 patient ccRCC tumors compared to their adjacent normal tissues. (E) qRT-PCR analysis of APOL1 mRNA in tumors of A-498, 786-O and Caki-1 cells. Actin served as an internal control. (F) Immunohistochemistry of APOL1 expression in tumors of 786-O and Caki-1 cells. (G) APOL1 expression by cancer stages in patient ccRCC tumors. (H) APOL1 protein expression by cancer grades in Clinical Proteomic Tumor Analysis Consortium (CPTAC) samples of ccRCC. [file Presentation_1.pptx]

## Slide 1
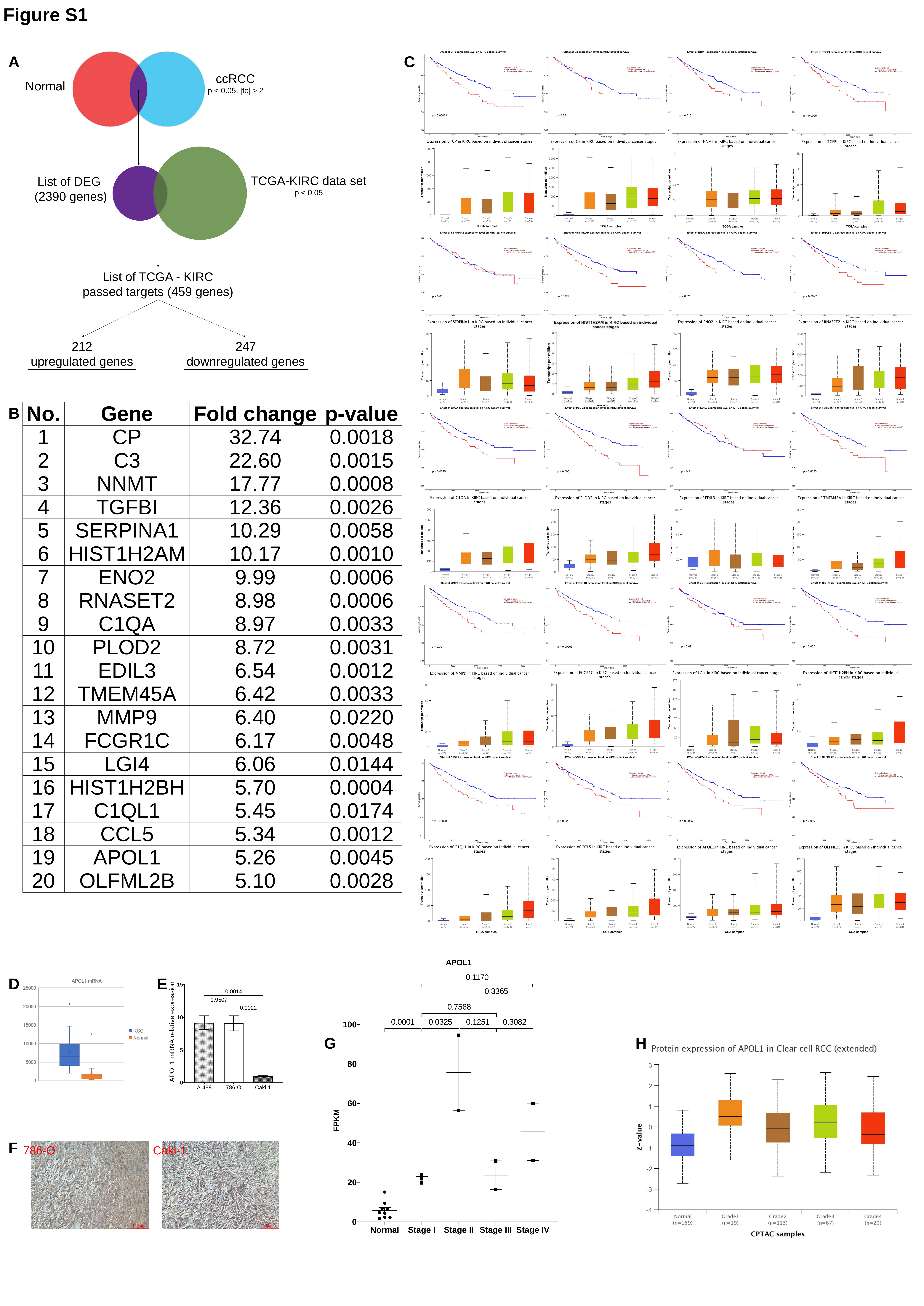

Figure S1
A
C
ccRCC
p < 0.05, |fc| > 2
Normal
TCGA-KIRC data set
p < 0.05
List of DEG
(2390 genes)
List of TCGA - KIRC
passed targets (459 genes)
212
upregulated genes
247
downregulated genes
B
| No. | Gene | Fold change | p-value |
| --- | --- | --- | --- |
| 1 | CP | 32.74 | 0.0018 |
| 2 | C3 | 22.60 | 0.0015 |
| 3 | NNMT | 17.77 | 0.0008 |
| 4 | TGFBI | 12.36 | 0.0026 |
| 5 | SERPINA1 | 10.29 | 0.0058 |
| 6 | HIST1H2AM | 10.17 | 0.0010 |
| 7 | ENO2 | 9.99 | 0.0006 |
| 8 | RNASET2 | 8.98 | 0.0006 |
| 9 | C1QA | 8.97 | 0.0033 |
| 10 | PLOD2 | 8.72 | 0.0031 |
| 11 | EDIL3 | 6.54 | 0.0012 |
| 12 | TMEM45A | 6.42 | 0.0033 |
| 13 | MMP9 | 6.40 | 0.0220 |
| 14 | FCGR1C | 6.17 | 0.0048 |
| 15 | LGI4 | 6.06 | 0.0144 |
| 16 | HIST1H2BH | 5.70 | 0.0004 |
| 17 | C1QL1 | 5.45 | 0.0174 |
| 18 | CCL5 | 5.34 | 0.0012 |
| 19 | APOL1 | 5.26 | 0.0045 |
| 20 | OLFML2B | 5.10 | 0.0028 |
E
D
G
H
F
786-O
Caki-1

## Slide 2
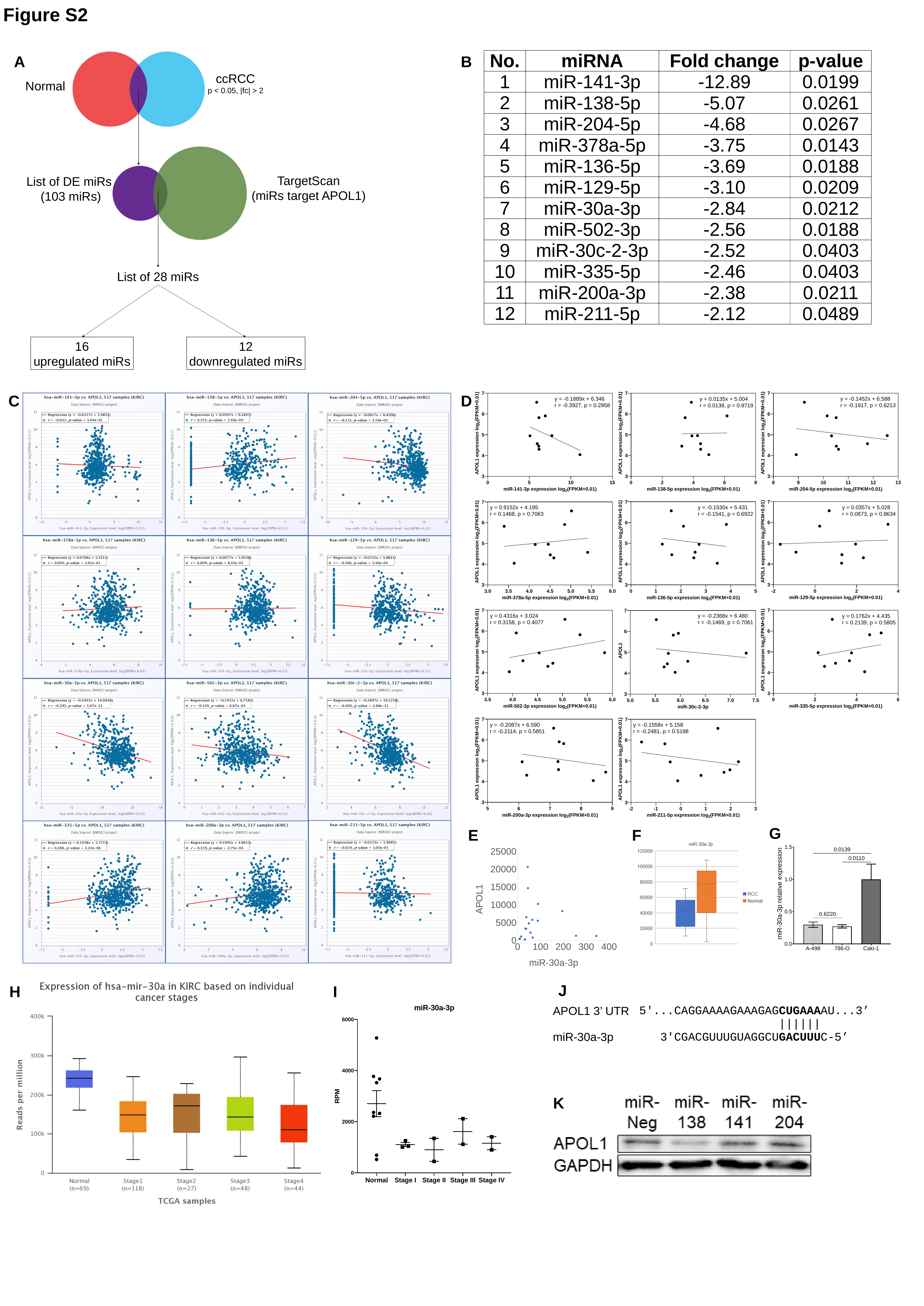

Figure S2
A
B
| No. | miRNA | Fold change | p-value |
| --- | --- | --- | --- |
| 1 | miR-141-3p | -12.89 | 0.0199 |
| 2 | miR-138-5p | -5.07 | 0.0261 |
| 3 | miR-204-5p | -4.68 | 0.0267 |
| 4 | miR-378a-5p | -3.75 | 0.0143 |
| 5 | miR-136-5p | -3.69 | 0.0188 |
| 6 | miR-129-5p | -3.10 | 0.0209 |
| 7 | miR-30a-3p | -2.84 | 0.0212 |
| 8 | miR-502-3p | -2.56 | 0.0188 |
| 9 | miR-30c-2-3p | -2.52 | 0.0403 |
| 10 | miR-335-5p | -2.46 | 0.0403 |
| 11 | miR-200a-3p | -2.38 | 0.0211 |
| 12 | miR-211-5p | -2.12 | 0.0489 |
ccRCC
p < 0.05, |fc| > 2
Normal
TargetScan
(miRs target APOL1)
List of DE miRs
(103 miRs)
List of 28 miRs
16
upregulated miRs
12
downregulated miRs
y = -0.1452x + 6.588
r = -0.1917, p = 0.6213
y = 0.0135x + 5.004
r = 0.0138, p = 0.9719
y = -0.1889x + 6.346
r = -0.3927, p = 0.2958
D
C
y = 0.0357x + 5.028
r = 0.0673, p = 0.8634
y = -0.1530x + 5.431
r = -0.1541, p = 0.6922
y = 0.9152x + 4.195
r = 0.1468, p = 0.7063
y = 0.4316x + 3.024
r = 0.3158, p = 0.4077
y = 0.1762x + 4.435
r = 0.2139, p = 0.5805
y = -0.2368x + 6.480
r = -0.1469, p = 0.7061
y = -0.2087x + 6.590
r = -0.2114, p = 0.5851
y = -0.1558x + 5.158
r = -0.2481, p = 0.5198
G
E
F
### Chart
| Category | ENSG00000100342.21|APOL1 |
|---|---|
J
H
I
| APOL1 3’ UTR miR-30a-3p | 5'...CAGGAAAAGAAAGAGCUGAAAAU...3’                     ||||||      3'CGACGUUUGUAGGCUGACUUUC-5’ |
| --- | --- |
K

## Slide 3
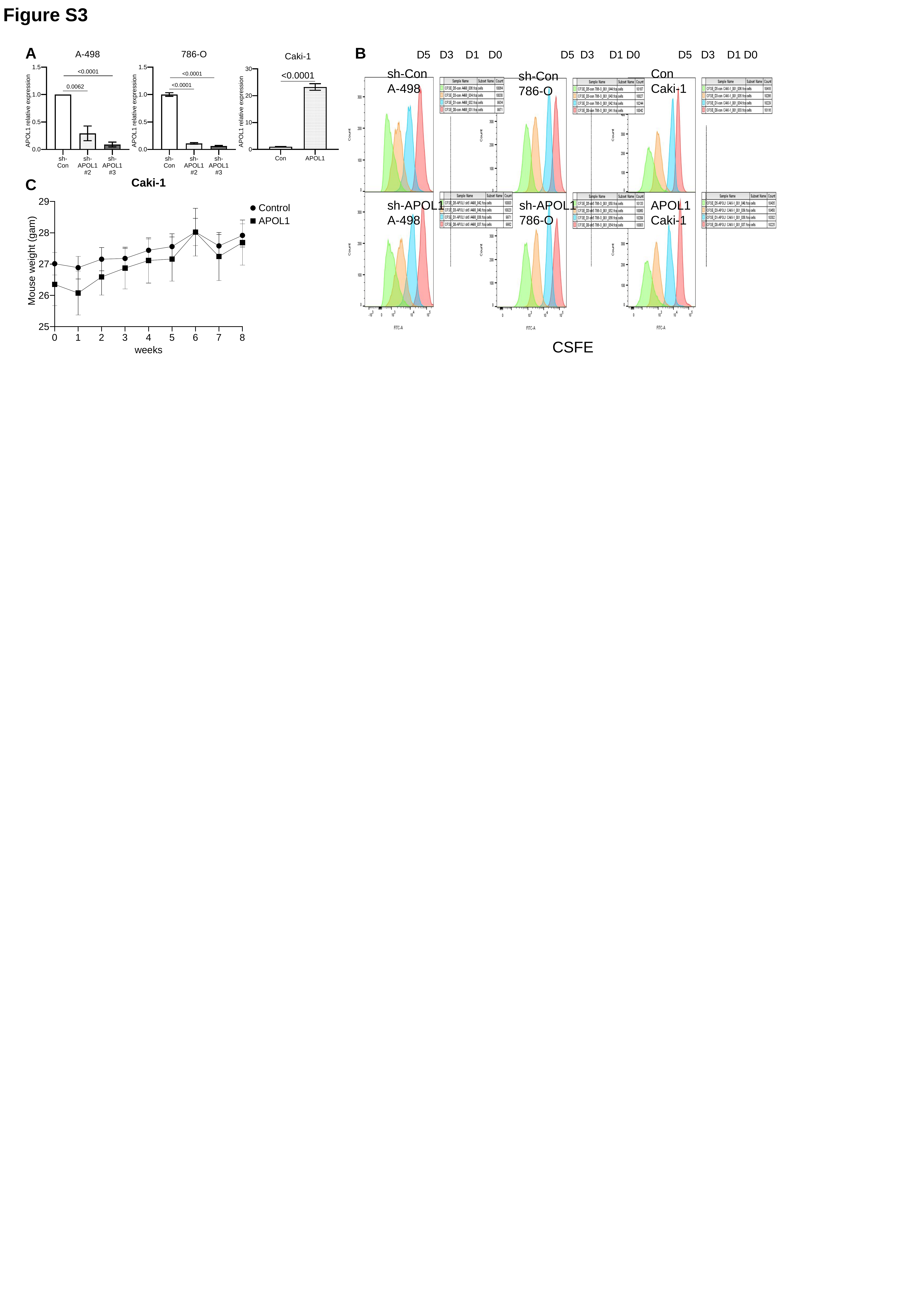

Figure S3
B
A
D5 D3 D1 D0
D5 D3 D1 D0
D5 D3 D1 D0
sh-Con
A-498
Con
Caki-1
sh-Con
786-O
sh-APOL1
A-498
sh-APOL1
786-O
APOL1
Caki-1
CSFE
C

## Slide 4
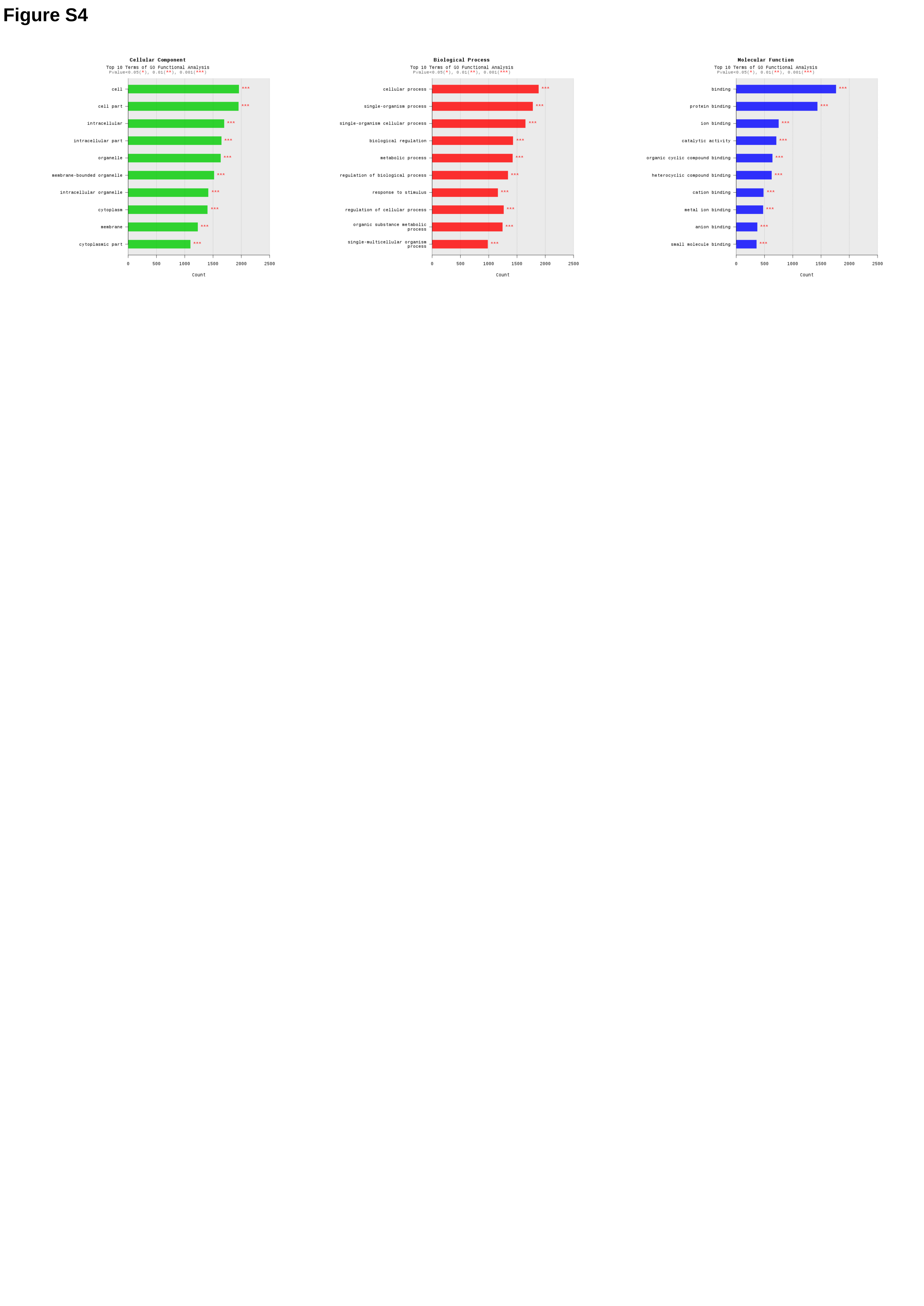

Figure S4
